# A Study on the Differences in Rumen Microbiota–Liver Gluconeogenesis–Mitochondrial Interaction Between Tibetan Sheep and Hu Sheep in the Qinghai–Tibet Plateau

**DOI:** 10.3390/ani15111603

**Published:** 2025-05-30

**Authors:** Qianling Chen, Yuzhu Sha, Xiu Liu, Min Gao, Xiaowei Chen, Wenxin Yang, Wei Huang, Jiqing Wang, Yapeng He, Xu Gao, Yanyu He

**Affiliations:** 1 Gansu Key Laboratory of Herbivorous Animal Biotechnology, College of Animal Science and Technology, Gansu Agricultural University, Lanzhou 730070, China; chenqianling223@163.com (Q.C.); shayz@st.gsau.edu.cn (Y.S.); gm12017101@163.com (M.G.); cxw20002022@163.com (X.C.); aaaaa0108@163.com (W.Y.); 18294737108@163.com (W.H.); wangjq@gsau.edu.cn (J.W.); 18894448066@163.com (Y.H.); gx2049879994@163.com (X.G.); 2 School of Fundamental Sciences, Massey University, Palmerston North 4410, New Zealand

**Keywords:** Hu sheep, Tibetan sheep, rumen microbiota, hepatic gluconeogenesis, mitochondrion

## Abstract

As high-quality sheep germplasm resources in China, Hu sheep are characterized by fast growth and development, high fecundity, and tolerance to drought and cold. Tibetan sheep, adapted to high-altitude environments, have developed strong environmental adaptability. To explore the differences in the interaction among rumen microbial flora, hepatic gluconeogenesis, and mitochondrial function between Tibetan sheep and Hu sheep in the Qinghai–Tibet Plateau, this study systematically compared and analyzed the rumen flora density, key enzyme activities related to hepatic gluconeogenesis and mitochondrial function, and the expression levels of related genes in Tibetan sheep and Hu sheep under identical feeding management conditions, followed by correlation analysis. The results showed that Hu sheep had significantly higher densities of *Ruminobacteramylophilus* (*Ram*) and *Fibrobacter succinogenes* (*Fs*) associated with starch and protein degradation (*p* < 0.01). The expression levels of *Forkhead box O1* (*FOXO1*), pyruvate carboxylase (PC) activity, and adenosine triphosphate (ATP) content were also significantly higher than those in Tibetan sheep (*p* < 0.01). In contrast, Tibetan sheep had higher densities of *Butyrivibrio fibrisolvens* (*Bf*), *Ruminococcus albus* (*Ra*), *Ruminococcus flavefaciens* (*Rf*), etc., related to cellulose degradation (*p* < 0.01). The gluconeogenesis-related genes, *Glucose-6-phosphatase catalytic subunit 1* (*G6PC1*) and *phosphoenolpyruvate carboxykinase1* (*PCK1*), and the activities of phosphoenolpyruvate carboxykinase (PEPCK) and fructose-1,6-bisphosphatase (FBPase) were significantly higher in Tibetan sheep than in Hu sheep (*p* < 0.01). Mitochondrial function-related genes *Mitofusin-1* (*Mfn1*), *Mitofusin-2* (*Mfn2*), *subunit 6 of ATP synthase* (*ATP6*), *cytochrome b* (*Cytb*), etc., also showed significantly higher expression in Tibetan sheep (*p* < 0.01). While no significant differences were observed in the contents of citric acid (CA), pyruvic acid (PA), glucose (Glu), etc. (*p* > 0.05). Correlation analysis indicated that rumen flora was associated with the key enzyme activities and gene expressions of hepatic gluconeogenesis and mitochondrial function to varying degrees. In summary, Tibetan sheep exhibit strong fiber degradation capacity, the efficient utilization of gluconeogenic intermediates, and mitochondrial oxidative phosphorylation (OXPHOS) ability, forming adaptive strategies for high-altitude environments. By contrast, Hu sheep show efficient protein and starch degradation capacity, thereby enhancing the supply of gluconeogenic precursors. It is indicated that when introducing Hu sheep to high-altitude areas, dietary intervention can be used to regulate rumen microorganisms, such as increasing fiber-decomposing bacteria or enhancing mitochondrial oxidative capacity, to counteract metabolic limitations induced by hypoxia.

## Simple Summary

This study explores how Tibetan sheep and Hu sheep adapt to the Qinghai–Tibet Plateau environment by comparing their rumen microbiota, hepatic gluconeogenesis, and mitochondrial function. Results show Tibetan sheep have higher abundances of fiber-degrading bacteria *Ruminococcus albus*, enhancing crude fiber digestion and gluconeogenic intermediate utilization via upregulated enzymes PEPCK and FBPase and mitochondrial genes *Mfn1* and *Mfn2*. Hu sheep excel in starch/protein degradation with higher *Ruminobacteramylophilus* and *Fibrobacter succinogenes*, boosting hepatic PC activity and gluconeogenic precursor supply. Correlation analysis reveals that rumen flora interacts with host metabolic pathways. The findings clarify adaptive strategies: Tibetan sheep rely on fiber digestion and mitochondrial oxidative phosphorylation, while Hu sheep optimize nutrient metabolism. For introducing Hu sheep to high altitudes, the dietary regulation of fiber-decomposing bacteria or mitochondrial function may alleviate hypoxic metabolic constraints.

## 1. Introduction

Environmental factors are the most important drivers of animal evolution. The environment shapes the genetic representations of the populations that inhabit it [1]. As a plateau-specific sheep breed, Tibetan sheep exhibit remarkable physiological characteristics of cold tolerance and hypoxia resistance. Through long-term adaptation to extremely high-altitude environments, they have evolved superior survival capabilities in hypoxic conditions. Hu sheep have excellent characteristics, such as fast growth, strong reproduction, early sexual maturity, and strong adaptability to the natural environment. In recent years, the breeding area of Hu sheep has gradually been introduced into the Qinghai–Tibet Plateau from the low altitude area [2], which helps to enrich the sheep germplasm resources of the Qinghai–Tibet Plateau. It also makes positive contributions to promoting the development of the sheep industry in the Qinghai–Tibetan Plateau.

As a distinctive digestive organ in ruminants, the rumen plays a pivotal role in their adaptation to high-altitude environments. The fermentation and digestion of feed in the rumen are mainly microbial communities [3,4]. According to their nutritional functions, rumen microorganisms can be classified into starch-degrading bacteria, fiber-degrading bacteria, protein-degrading bacteria, etc. [5]. During the process of adapting to the environment, there may be certain variations in these bacterial floras among different species or breeds. Studies showed that with the increase in the external environmental temperature, the number of Streptococcus bovis and starch-degrading bacteria in the rumen of Holstein cows was elevated. However, in sheep and goats, the number of starch-degrading bacteria in the rumen decreases [6]. Liu et al. [7] found that heat stress significantly inhibits the growth of *Ruminalococci*, *Ruminalococci* spp., and *Streptococcus* spp. in dairy cows and reduces the body’s ability to degrade the diet, which leads to an insufficient supply of energy. Glucose, as an energy carrier, is involved in the metabolism and synthesis pathway of all types of mammal cells [8,9]. Therefore, glucose synthesis is particularly important when animals are under-supplied with energy.

Hepatic gluconeogenesis can provide ruminants with more than 80% of their glucose requirements [10], so regulating ruminant gluconeogenesis is an effective means of improving their health, growth, and production performance. The main precursors for gluconeogenesis in ruminants include propionic acid, glycerol, amino acids, and lactic acid [11]. The key factors influencing hepatic gluconeogenesis are the concentration of effective glycogen substrates and the activity of several regulatory enzymes. Phosphoenolpyruvate carboxykinase (PEPCK) is one of the rate-limiting steps of catalytic gluconeogenesis, catalyzing the reaction of the decarboxylation of oxaloacetate to generate phosphoenolpyruvate. Glucose-6-phosphatase (G6Pase), the rate-limiting enzyme in the final step of gluconeogenesis, catalyzes the hydrolysis of glucose 6-phosphate into free glucose. Phosphoenolpyruvate carboxykinase (PEPCK) and G6Pase activities are modulated through both transcriptional and post-transcriptional regulatory mechanisms [12]. It was found that *FOXO1* can promote gluconeogenesis G6Pase and PEPCK expression [13]. Genes encoding *Glucose-6-phosphatase catalytic subunit 1* (*G6PC1*) and *phosphoenolpyruvate carboxykinase1*, *2* (*PCK1*, *PCK2*) are key determinants of gluconeogenesis initiation [14]. In addition, the hepatic gluconeogenesis in ruminants relies on mitochondrial energy supply.

Mitochondria are an important site for aerobic respiration in all cells, capable of generating ATP, maintaining cellular Ca^2+^ homeostasis, etc. ATP produced by cellular mitochondria is involved in the oxidation of substrates in glycolysis, the tricarboxylic acid cycle (TCA), and pyruvate decarboxylation [15]. ATP, as a major source of energy for the metabolism of living organisms, plays an important role in the respiratory chain of mitochondria. As the marker enzymes of mitochondria, the strength of succinate dehydrogenase (SDH) and cytochrome oxidase (COX) activities reflects the degree of the aerobic metabolism of the cell [16]. Once the mitochondria are damaged, a series of cascade reactions will occur and cause diseases. Therefore, maintaining normal mitochondrial biosynthesis and function and balancing the mitochondrial dynamics are important prerequisites for the cells to carry out normal aerobic respiration. Physiologically, *PGC-1α* is one of the important regulators of mitochondrial biosynthesis [17] and significant mitochondrial damage occurs after the specific knockdown of the mouse Peroxisome proliferator-activated receptor-gamma coactivator-1-alpha (PGC-1α) protein [18]. Mitochondrial dynamics homeostasis is regulated by Mitofusin-1 (Mfn1), Mitofusin-2(Mfn2), Optic atrophy (OPA1), Mitochondrial fission 1 (Fis1), and Mitochondrial fission factor (MFF) proteins. Aberrant expression of the regulatory proteins causes an imbalance in mitochondrial dynamics and leads to disease occurrence [19]. Studies have revealed that mitochondria share the same structural and functional properties with gut flora prokaryotes. The two may also play their unique physiological roles through certain linkages [20,21]. Host mitochondria can modulate gut microbiota diversity by releasing Reactive Oxygen Species (ROS) [22] and gut flora metabolites, in turn, inducing functional alterations in mitochondria. Therefore, we used the variation patterns of rumen flora density in Tibetan sheep and Hu sheep under identical feeding and management conditions as the research entry point. This study reveals the differences in the interaction among rumen microbial flora, hepatic gluconeogenesis, and mitochondria between Tibetan sheep and Hu sheep through correlation analysis with the expression levels of hepatic gluconeogenesis-related genes, key enzyme activities, and mitochondrial functions. This study also provides a theoretical basis for the introduction and adaptation of Hu sheep to the alpine environment of the Qinghai–Tibet Plateau.

## 2. Materials and Methods

### 2.1. Inclusion and Exclusion Criteria

All animal experiments were conducted in strict compliance with the regulations for the Administration of Affairs Concerning Experimental Animals (Ministry of Science and Technology, China; revised in June 2004). Sample collection protocols were approved by the Livestock Care Committee of Gansu Agricultural University (Approval No. GAU-LC-2020-27).

### 2.2. Experimental Animals and Sample Collection

The test samples were collected from a single herd managed by the same herder in Nawu Town, Hezuo City, Gannan Tibetan Autonomous Prefecture, Gansu Province (with an altitude of 3000 m, longitude 103° E, latitude 35° N). The sampling was conducted in the middle of September 2023. All the experimental samplings were carried out with the consent of the sheep owners. Six Tibetan sheep and six Hu sheep were chosen, each with a mean body weight of 34 kg and a mean age of 1 year old. The test sheep had normal feeding and rumination behaviors. Their coats were shiny, their body conditions were good, and their feces were oval-shaped and adhered to each other after falling to the ground, indicating that they were in good health. All sheep were managed under the local traditional natural grazing system. They were herded in a fixed pasture from 8 a.m. to 7 p.m. daily without supplemental feeding, allowing free foraging on the pasture. Nutritional compositions of the pasture grass are presented in Table S1.

Before morning grazing, rumen contents were collected using a gastric tube-type rumen sampler for sheep. Three tubes of rumen fluid were collected from each sheep. The collected rumen fluid was rapidly placed into a liquid nitrogen tank for freezing, then transported back to the laboratory and stored at −80 °C for subsequent DNA extraction. Subsequently, the sheep were euthanized using the local traditional method (cervical vein bloodletting). The liver was immediately dissected, and a liver tissue block (1 cm^2^) was collected and placed into a cryopreservation tube, rapidly immersed in liquid nitrogen and stored for subsequent total RNA extraction and enzyme activity assays.

### 2.3. Liver Tissue Gluconeogenesis and Mitochondrial Function Measurements

Liver Pyruvate carboxylase, phosphoenolpyruvate carboxykinase, Fructose 1, 6-bisphosptase, and Glucose-6-phosphatase activities were measured using sheep Pyruvate carboxylase (kit number was PC-1-Y), phosphoenolpyruvate carboxykinase (kit number was PEPCK-1-Y), Fructose 1, 6-bisphosptase (kit number was FBP-1-Y), and Glucose-6-phosphatase (kit number was G6P-1-Y) ELISA kits. The mitochondrial adenosine triphosphate, citric acid, and pyruvic acid content of Tibetan and Hu sheep were determined by using sheep adenosine triphosphate (kit number was FHTE-1-Y), citric acid (kit number was CA-1-W), and pyruvic acid (kit number was PA-1-Y) content kits (micro method), and respiratory chain complex IV activity was determined by using a sheep mitochondrial complex IV (kit number was FHTD-1-Y) kit. All procedures were strictly performed according to the instructions of the reagent kits. The aforementioned detection kits were purchased from Suzhou Keming Biotechnology Co., Ltd. The liver glucose content was measured using glucose content test kits (kit number was ml076790) from Shanghai Enzyme-linked Biotechnology Co. Instrument: Spectrophotometer (Thermo 3020, Thermo Fisher Scientific, Shanghai, China). Standard curve and content calculation: in the Excel 2021 worksheet. The standard linear regression curve was plotted with the standard concentration as the abscissa and the corresponding sample OD values as the ordinate. The concentration and content of each liver sample were calculated using the curve equation.

### 2.4. Total RNA Extraction and Gene Expression Measurement in Liver Tissue

Total liver RNA of Tibetan sheep and Hu sheep was extracted using the Trizol reagent method (DP762-T1C; Life Technologies, Carlsbad, CA, USA). The concentration and purity of RNA were determined by an ultra-micro spectrophotometer (Therm Nano Drop-2000; Thermo Scientific, Waltham, MA, USA), and the ratio of OD260:OD280 was in the range of 1.8–2.1, indicating high-purity RNA extraction. RNA integrity was detected using an agarose gel electrophoresis instrument (Agient2100, LabChip GX). A single and distinct band was observed via the agarose gel imaging system. (Ready Agarose, Bio-Rad, Hercules, CA, USA), confirming intact RNA structure. cDNA synthesis was carried out using a reverse transcription kit (HiScript^®^ II Q RT SuperMix for qPCR; Nanjing, China). β-actin served as the internal reference gene. Primers were designed via NCBI BLAST (https://www.ncbi.nlm.nih.gov/tools/primer-blast, accessed on 8 October 2024), and their specificity was validated using Nucleotide BLAST (https://blast.ncbi.nlm.nih.gov/Blast.cgi, accessed on 8 October 2024) and DNAMAN 8 software. Primers were synthesized by Beijing AuGCT Biotech Co., Ltd., Beijing, China, with gene sequences detailed in Table 1 and Table 2. Relative expression levels of genes were measured using an Applied Biosystems Q6 real-time fluorescent quantitative PCR instrument, employing a 20 μL reaction system: a 10 μL Mix enzyme, 0.4 μL each of forward and reverse primers, 2 μL of template DNA, and 7.2 μL of ddH_2_O. The reaction conditions were as follows: pre-denaturation at 95 °C for 30 s; cyclic reaction at 95 °C for 10 s and 60 °C for 30 s, for 40 cycles; melting curve analysis (95 °C for 15 s, 60 °C for 60 s, 95 °C for 15 s). Relative gene expression levels were calculated by normalizing to β-actin as the internal reference gene, with data analysis performed using the 2^−∆∆CT^ method [23].

### 2.5. Total DNA Extraction of Rumen Microorganisms and Determination of Bacterial Population Density

Total DNA from rumen microbiota of Tibetan sheep and Hu sheep was extracted using the Jian Shi Biological Fecal DNA Extraction Kit. OD260/280 value was detected by super differential photometer (Thern Nano Drop-2000).

*Ruminococcus flavefaciens*, *Ruminococcus albus*, *Fibrobacter succinogenes*, *Selenomonas ruminantium*, *Treponema bryantii*, *Clostridium butyricum*, *Ruminobacteramylophilus*, and *Butyrivibrio fibrisolvens* were selected for relative quantitative studies using rumen microbial DNA as templates, respectively. NCBI BLAST was used to search for bacterial sequences and design primers (Table 3), which were synthesized by Beijing Aoke Dingsheng Biotechnology Co., Beijing, China. The microbiota density was determined using an Applied Biosystems Q6 real-time fluorescent quantitative PCR instrument, with the reaction system and conditions consistent with those described for gene expression analysis. Bacterial internal references were used for calibration to calculate the microbiota density.

### 2.6. Statistical Analysis of the Data

The experimental data were collated using Excel 2016 and analyzed with SPSS 24.0 software. All analyzed data are presented as “mean ± standard error”, with a statistical significance level set at *p* < 0.05. Based on the previous research on the rumen microorganisms of Tibetan sheep and Hu sheep under the same feeding and management conditions [24], this study conducted a Spearman analysis on the rumen microorganisms, the activities of key gluconeogenic enzymes and their gene expression levels, and the indicators related to mitochondrial function in Tibetan sheep and Hu sheep, and mitochondrial function-related indexes in Tibetan sheep and Hu sheep were correlated between them, and the correlation analyses were all performed using the Spearman correlation test with a screening criterion of *p* < 0.05.

## 3. Results

### 3.1. Differences in Rumen Flora Density Between Tibetan Sheep and Hu Sheep

**Figure 1:**
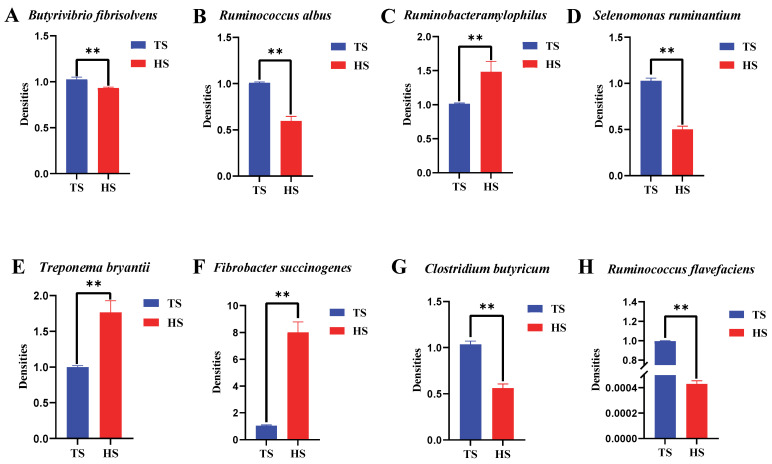
Rumen microbiota density of Tibetan sheep and Hu sheep. Note: ** *p* < 0.01.

Significant differences were observed in the densities of each bacterial group in the rumen of Tibetan sheep and Hu sheep (Figure 1). The densities of *Ruminobacteramylophilus*, *Treponema bryantii*, and *Fibrobacter succinogenes* in Hu sheep were significantly higher than those in Tibetan sheep (*p* < 0.01), while the densities of *Butyrivibrio fibrisolvens*, *Ruminococcus albus*, *Selenomonas ruminantium*, *Clostridium butyricum*, and *Ruminococcus flavefaciens* in Tibetan sheep were significantly higher than those in Hu sheep (*p* < 0.01).

### 3.2. Determination of Hepatic Glyoxylase Activity and Related Gene Expression in Tibetan Sheep and Hu Sheep

**Figure 2:**
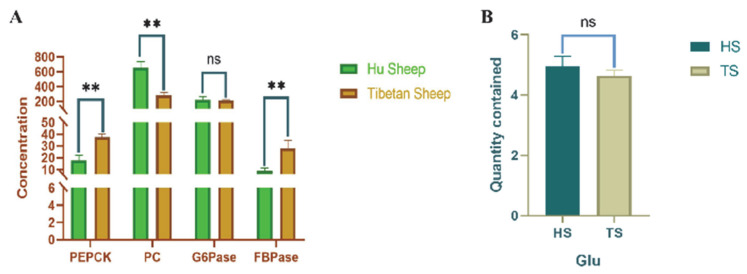
Analysis of hepatic gluconeogenesis pathways in Tibetan sheep and Hu sheep. (**A**) Concentrations of key enzymes in the livers of Tibetan sheep and Hu sheep. (**B**) Liver glucose content in Tibetan sheep and Hu sheep. Note: ** *p* < 0.01; ns *p* > 0.05.

**Figure 3:**
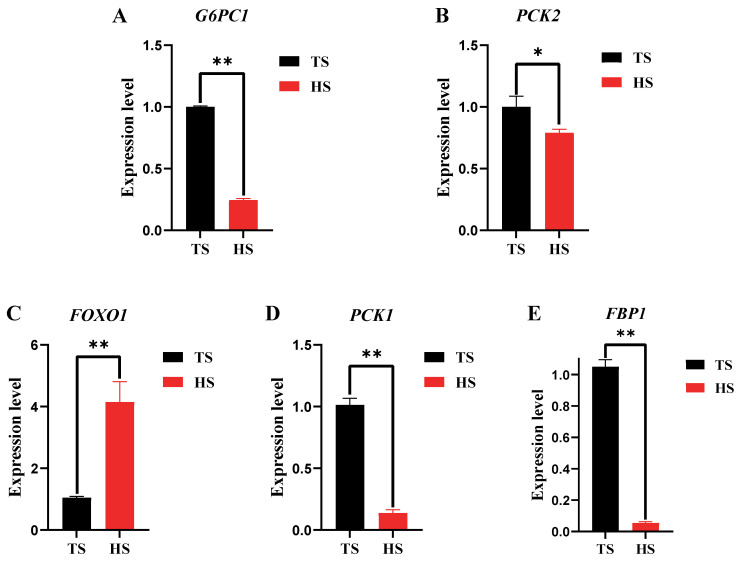
Expression of genes related to hepatic gluconeogenesis in Tibetan sheep and Hu sheep. Note: ** *p* < 0.01; * *p* < 0.05.

Liver gluconeogenic enzyme activity and glucose content of Tibetan and Hu sheep (Figure 2) show that PC activity was the highest in both Tibetan and Hu sheep livers. PEPCK and FBPase activities were significantly higher in Tibetan sheep than in Hu sheep (*p* < 0.01), while PC activity was significantly higher in Hu sheep than in Tibetan sheep (*p* < 0.01). The difference between G6Pase activity, as well as glucose content, was not significant in both Tibetan and Hu sheep (*p* > 0.05). The results of gluconeogenesis-related gene expression in Tibetan and Hu sheep are shown in Figure 3. The expression level of the *FOXO1* gene in Hu sheep was extremely significantly higher than that in Tibetan sheep (*p* < 0.01). The expression levels of *G6PC1*, *FBP1*, and *PCK1* genes in Tibetan sheep were all extremely significantly higher than those in Hu sheep (*p* < 0.01), and the expression level of the *PCK2* gene in Tibetan sheep was significantly higher than that in Hu sheep (*p* < 0.05).

### 3.3. Determination of Mitochondrial Function in Tibetan Sheep and Hu Sheep

**Figure 4:**
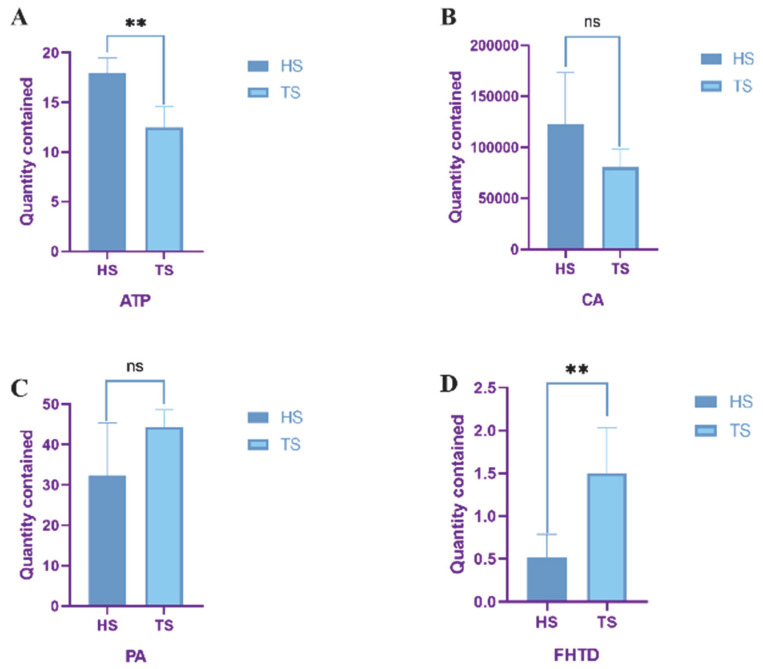
Concentrations of key mitochondrial enzymes in Tibetan sheep and Hu sheep. From (**A**–**D**) are the contents of ATP, CA, PA and FHTD. Note: ** *p* < 0.01; ns *p* > 0.05.

**Figure 5:**
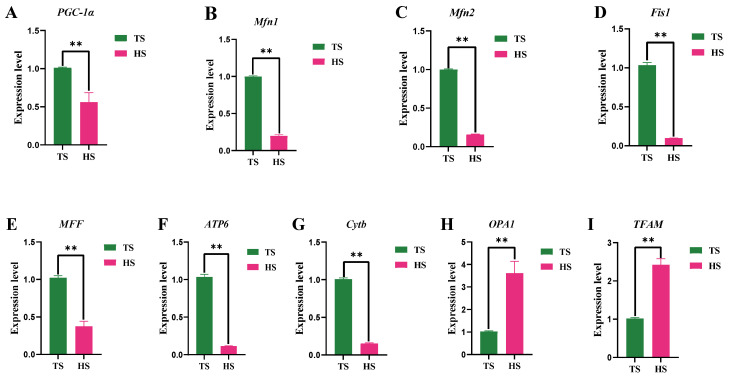
Expression of mitochondria-related genes in Tibetan sheep and Hu sheep. Note: ** *p* < 0.01.

The mitochondrial enzyme activities and intermediate contents of Tibetan and Hu sheep are shown in Figure 4. The ATP content of Hu sheep was significantly higher than that of Tibetan sheep (*p* < 0.01), whereas the FHTD activity of Tibetan sheep was significantly higher than that of Hu sheep (*p* < 0.01). The differences between the two species of sheep in the CA and PA contents were not significant (*p* > 0.05). The histogram of mitochondrial gene expression (Figure 5) showed that the expression of *OPA1* and *TFAM* genes in Hu sheep were extremely significantly higher than those in Tibetan sheep (*p* < 0.01), while the expression levels of *Mfn1*, *Mfn2*, *Fis1*, *MFF*, *ATP6*, *Cytb*, and P*GC-1α* genes in Tibetan sheep were extremely significantly higher than those in Hu sheep (*p* < 0.01).

### 3.4. Correlation Analysis of Rumen Microbial–Hepatic Gluconeogenesis–Mitochondrial Function

**Figure 6:**
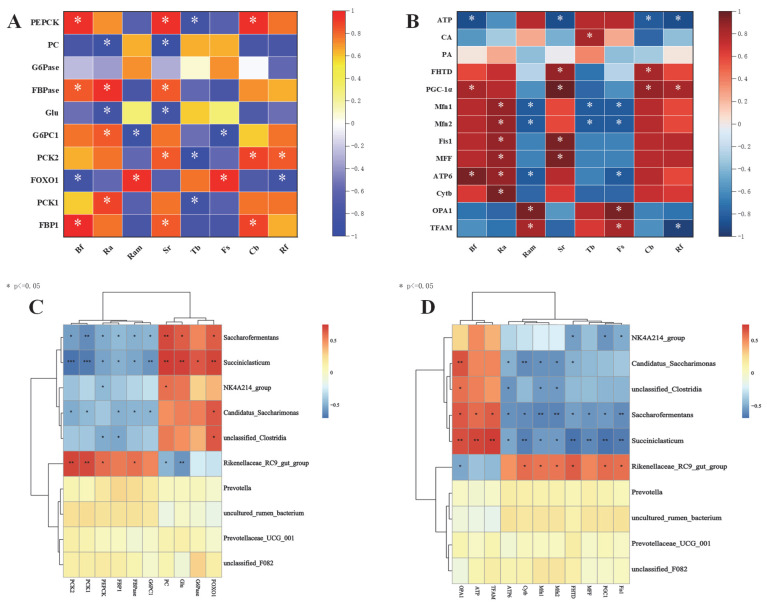
Heat map of rumen microbiota, key enzymes, and genes involved in gluconeogenesis, and mitochondrial function correlation in Tibetan and Hu sheep. (**A**) Correlation heatmap of rumen flora with key gluconeogenic enzyme activities and their gene expression levels. (**B**) Correlation heatmap of rumen flora with mitochondrial function indexes. (**C**) Correlation heatmap of the top ten microbial genera in rumen abundance with key gluconeogenic enzyme activities and their gene expression levels. (**D**) Correlation heatmap of the top ten microbial genera in rumen abundance with mitochondrial function indexes. Note: Correlation heatmap * *p* < 0.05, ** *p* < 0.01, *** *p* < 0.001.

The correlations between rumen microorganisms and the activity of key enzymes for gluconeogenesis and their gene expression are shown in Figure 6A,C. Among them, the *FOXO1* gene was significantly positively correlated with *Ruminobacteramylophilus*, *Fibrobacter succinogenes*, *Succiniclasticum*, *Candidatus-Saccharimonas*, and *Saccharofermentans* (*p* < 0.05) and significantly negatively correlated with *Butyrivibrio fibrisolvens* and *Ruminococcus flavefaciens*. The *PCK2* and *PCK1* genes showed negative correlations of varying degrees with *Treponema bryantii*, *Succiniclasticum*, *Candidatus-Saccharimonas*, and *Saccharofermentans* (*p* < 0.05). The *PCK1* and *PCK2* genes were significantly positively correlated with the *Rikenellaceae-RC9-gut-group*, and the *PCK2* gene was significantly positively correlated with *Selenomonas ruminantium*, *Clostridium butyricum*, and *Ruminococcus flavefaciens* (*p* < 0.05). The *G6PC1* gene was significantly negatively correlated with *Ruminobacteramylophilus*, *Fibrobacter succinogenes*, and *Succiniclasticum* (*p* < 0.05). The *FBP1* gene was significantly positively correlated with *Butyrivibrio fibrisolvens*, *Selenomonas ruminantium*, and *Clostridium butyricum* (*p* < 0.05) but significantly negatively correlated with *Succiniclasticum* and *Saccharofermentans* (*p* < 0.05). PEPCK was significantly negatively correlated with *Ruminococcus albus*, *Treponema bryantii*, and *Candidatus-Saccharimonas* (*p* < 0.05). The content of Glu and PC activity was significantly negatively correlated with *Ruminococcus albus*, *Selenomonas ruminantium*, and the *Rikenellaceae-RC9-gut-group* (*p* < 0.05) but significantly positively correlated with *Succiniclasticum* and *Saccharofermentans* (*p* < 0.05). FBPase was significantly positively correlated with *Butyrivibrio fibrisolvens*, *Selenomonas ruminantium*, *Ruminococcus albus*, and the *Rikenellaceae-RC9-gut-group* (*p* < 0.05). PEPECK was significantly positively correlated with *Butyrivibrio fibrisolvens*, *Selenomonas ruminantium*, *Clostridium butyricum*, and the *Rikenellaceae-RC9-gut-group* (*p* < 0.05) but significantly negatively correlated with *Succiniclasticum* and *Saccharofermentans* (*p* < 0.05).

The results of the correlation analysis of rumen microorganisms and indicators related to mitochondrial function in Tibetan sheep and Hu sheep are shown in Figure 6B,D. *Ruminococcus albus* was significantly positively correlated with the *ATP6*, *MFF*, *Fis1*, *Mfn1*, and *Mfn2* genes (*p* < 0.05). *Ruminobacteramylophilus* and *Selenomonas ruminantium* were significantly positively correlated with the *PGC-1α*, *OPA1*, and *Fis1* genes (*p* < 0.05). *Ruminobacteramylophilus* and *Fibrobacter succinogenes* were significantly negatively correlated with the *ATP6*, *Mfn1*, and *Mfn2* genes (*p* < 0.05). *Selenomonas ruminantium* was significantly positively correlated with the *MFF*, *Fis1*, and *PGC-1α* genes and FHTD activity (*p* < 0.05) and significantly negatively correlated with ATP content (*p* < 0.05). *Treponema bryantii* was significantly negatively correlated with the *MFF* and *Mfn1* genes (*p* < 0.05), while being significantly positively correlated with CA content (*p* < 0.05). *Butyrivibrio fibrisolvens* and *Ruminococcus flavefaciens* were significantly positively correlated with the *PGC-1α* gene (*p* < 0.05) and significantly negatively correlated with ATP content (*p* < 0.05). Additionally, the *Rikenellaceae-RC9-gut-group* was significantly positively correlated with the *Cytb*, *Mfn2*, *Mfn1*, and *PGC-1α* genes as well as FHTD activity (*p* < 0.05), while being significantly negatively correlated with the *OPA1* gene (*p* < 0.05). *Succiniclasticum* and *Saccharofermentans* were extremely significantly positively correlated with the *TFAM* and *OPA1* genes as well as ATP content (*p* < 0.01), while *Succiniclasticum* was extremely significantly negatively correlated with genes such as *Cytb*, *MFF*, and *Fis1* (*p* < 0.01). *Saccharofermentans* was significantly negatively correlated with *ATP6*, *Cytb*, *MFF*, *PGC-1α*, etc. (*p* < 0.05). *Candidatus-Saccharimonas* was significantly positively correlated with *OPA1* (*p* < 0.05) and significantly negatively correlated with *Mfn2*, *ATP6*, *Mfn1*, etc. (*p* < 0.05).

## 4. Discussion

This study aims to compare the differences between Tibetan sheep and Hu sheep by analyzing the rumen microbial density, key enzyme activities related to liver gluconeogenesis and mitochondrial function, and the expression levels of related genes under the same feeding and management conditions so as to provide some ideas for the regulation of Hu sheep introduced into high-altitude areas. The rumen microbiota plays a crucial role in food digestion, growth and development, energy balance, and immune regulation in ruminant animals. *Fibrobacter succinogenes*, *Ruminococcus albus*, and *Ruminococcus flavefaciens* are the three major cellulose-degrading bacteria found in the rumen and are key members of the rumen microbiota in ruminant animals, with the ability to degrade cellulose and produce VFAs [25,26]. This study showed that under the same feeding and management conditions, the relative densities of *Ruminococcus flavefaciens* and *Ruminococcus albus* in Tibetan sheep were significantly higher than those in Hu sheep, while the relative density of *Fibrobacter succinogenes* was higher in Hu sheep. These results may be attributed to the differences in the genetic backgrounds of different breeds, leading to variations in the density of rumen microbial flora. Additionally, there are a wide variety and large number of microorganisms residing in the rumen, with complex interactions occurring among its members. Yeoman et al. [27] found that in vivo competition among these three main cellulose-degrading bacteria can also lead to this result. Except for the above-mentioned bacteria, the densities of *Ruminobacteramylophilus* and *Treponema bryantii* in Hu sheep were significantly higher than those in Tibetan sheep, while the densities of *Selenomonas ruminantium*, *Clostridium butyricum*, and *Butyrivibrio fibrisolvens* were higher in Tibetan sheep. *Ruminobacteramylophilus* is a major starch-decomposing bacterium, which plays an extremely important role in the digestion of protein, starch, and other feeds in ruminants [28]. Treponema *bryantii* produces formic acid, acetic acid, and succinic acid by fermenting pectin, cellobiose, sucrose, and monosaccharides [29], indicating that Hu sheep have a stronger decomposition of protein, starch, and monosaccharides than Tibetan sheep. As an important bacterial community in the rumen, *Selenomonas ruminantium* accounts for 51% of the total number of viable bacteria in the rumen, which can further convert the produced lactic acid into propionic acid [30] and can also use glycerol and other substances to produce propionic acid [31]. The remaining *Butyrivibrio fibrisolvens* and *Clostridium butyricum* mainly degrade lignocellulose to produce butyric acid, acetic acid, etc. [32], suggesting that the intestinal microecology evolved by Tibetan sheep in the plateau environment has a high digestibility of roughage and strong roughage tolerance.

In addition, this study further explored the hepatic gluconeogenesis process and mitochondrial function. Hepatic gluconeogenesis is the process by which the liver converts a variety of non-sugar substances, such as lactic acid, pyruvic acid, amino acids, and glycerol, into glucose or glycogen. This process is crucial for the body to utilize non-sugar substances to replenish blood glucose when there is an increase in energy demand or a shortage of glycogen reserves. Glucose production in ruminants mainly comes from hepatic gluconeogenesis, in which the body converts carbohydrates to VFAs through fermentation by rumen microorganisms. Key substances, such as propionic acid, are converted to glucose through hepatic gluconeogenesis. Amino acids can also be converted through this process to meet the energy requirements for growth [33]. Glycolytic activity is transcriptionally regulated by a variety of rate-limiting enzymes, including G6Pase, FBPase, PEPCK, and PC [34]. In this study, PC activity was significantly higher in Hu sheep than in Tibetan sheep. *Succiniclasticum* showed a significant positive correlation with PC activity. The level of enzyme activity determines the degree of hepatic gluconeogenesis. PC is a metabolic enzyme that participates in the first step of gluconeogenesis and catalyzes two consecutive reactions, carboxylating pyruvic acid to oxaloacetic acid [35]. Propionic acid, as one of the important components of VFAs, is the most important substrate for gluconeogenesis [36]. Rumen *Succiniclasticum* is a rumen bacterium that converts succinate to propionate [37]. Previous studies have shown that the concentration of propionic acid was significantly higher in Hu sheep than in Tibetan sheep (Table S2), suggesting that the concentration of gluconeogenic substrates was higher in Hu sheep than in Tibetan sheep, resulting in higher PC activity in Hu sheep, while there was higher FBPase and PEPCK activity Tibetan sheep. *Succiniclasticum* and PEPCK activity is an extremely significant negative correlation, ruminant body oxaloacetate, in addition to pyruvic acid production, but also through the tricarboxylic acid cycle malic acid regeneration and aspartic acid. The Tibetan sheep live long term in the high-altitude, low-oxygen, pasture grass of poor quality in the plateau environment. The formation of the alpine climate adapted to the special biological habits and digestive and metabolic mechanisms, the body of the demand for energy and oxygen is higher, leading to higher activity of the tricarboxylic acid cycle and the production of more oxaloacetic acid. The highly active PEPCK in Tibetan sheep converts oxaloacetate to phosphoenolpyruvate (PEP) [38], which is phosphorylated to produce F1,6BP, which is broken down to fructose 6-phosphate and inorganic phosphate catalyzed by FBPase and inorganic phosphate [39], which in turn is catalyzed by fructose isomerase to produce glucose 6-phosphate, and finally, G6Pase catalyzes the gluconeogenesis pathway to produce glucose [40], which is used to satisfy the glycemic requirements of Tibetan sheep in the plateau environment. In this study, there was no significant difference in glucose content between Tibetan sheep and Hu sheep. This indicates that Hu sheep can generate more propionic acid and other gluconeogenic substrates through the efficient degradation of starch, etc., and have higher liver PC activity, which may endow them with stronger gluconeogenic precursor supply capacity. Although the production of propionic acid in Tibetan sheep may be lower, the activities of PEPCK and FBPase are higher, which can accelerate the metabolism of gluconeogenic intermediates to provide the energy required for the plateau environment, and finally achieve a balance in glucose production with Hu sheep.

Glycolysis activity is regulated by a variety of transcription factors and genes, in addition to a variety of rate-limiting enzymes. *FOXO1*, a member of the O subclass of the forkhead transcription factor family, serves as an important transcriptional regulator in hepatic gluconeogenesis. It promotes glucose production by activating the expression of PEPCK and G6Pase in gluconeogenesis [41]. In this study, the expression level of the *FOXO1* gene in Hu sheep was significantly higher than that in Tibetan sheep, and it was significantly positively correlated with *Ruminobacteramylophilus*, *Fibrobacter succinogenes*, *Candidatus-Saccharimonas*, etc. *Candidatus-Saccharimonas* can increase the concentrations of fecal and serum acetate. Acetate can also be absorbed by the liver and used as a substrate for gluconeogenesis, and it can mediate liver protection by regulating glucose metabolism, reducing the levels of glucotoxic products and improving mitochondrial function [42], indicating that gut microbes are crucial for maintaining body energy balance and hepatic metabolic functions, including hepatic gluconeogenesis, glucose tolerance, etc. [43]. As a key gene of gluconeogenesis, *G6PC1* plays a role by mediating the terminal steps of the gluconeogenesis and glycogenolysis pathways [14]. *G6PC1* is significantly positively correlated with *Ruminococcus albus*, which can effectively degrade forage to produce VFAs, etc. VFAs are directly absorbed through the ruminal epithelium, transported to the blood, and undergo gluconeogenesis. In this way, the host can rapidly obtain blood glucose, and microorganisms are not inhibited by the end products [44]. In addition, *PCK1* and *PCK2* genes can also regulate hepatic gluconeogenesis activity, which mainly acts by encoding PEPCK. In this study, the expression of *PCK1* and *PCK2* genes was significantly higher in Tibetan sheep than in Hu sheep, and *PCK1* and *PCK2* were significantly and positively correlated with *Ruminococcus albus*, *Selenomonas ruminantium*, *Clostridium butyricum*, and the *Rikenellaceae-RC9-gut-group*. *PCK1* can block excessive glycolysis and promote gluconeogenesis by inhibiting the upregulation of the rate-limiting enzyme of excessive glycolysis HK2 [45]. Frazier et al. [43] found that *PCK2* may be the key gene in liver and microbe-mediated effects on gluconeogenesis. The *Rikenellaceae-RC9-gut-group* plays an important role in plateau animals. In wild mice in high-latitude regions, the abundance of the *Rikenellaceae-RC9-gut-group* is positively correlated with body weight. The flora helps adapt to the alpine environment by enhancing the production of VFAs [46]. This indicates that Tibetan sheep maintain blood glucose homeostasis by improving the efficiency of the gluconeogenic pathway through rumen flora and gluconeogenesis-related genes, so as to meet the gluconeogenic demand under the plateau’s low-quality feed.

**Figure 7:**
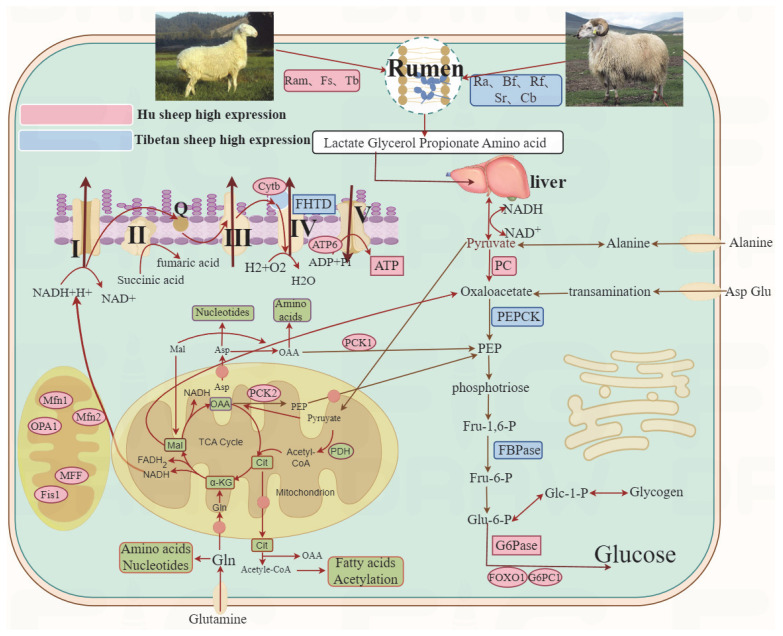
Diagram of the rumen microbiota–hepatic gluconeogenesis-–-mitochondrial interaction model. Note: *Ra*: *Ruminococcus albus*, *Ram*: *Ruminobacteramylophilus*, *Sr*: *Selenomonas ruminantium*, *Tb*: *Treponema bryantii*, *Fs*: *Fibrobacter succinogenes*, *Bf*: *Butyrivibrio fibrisolvens*, *Cb*: *Clostridium butyricum*, *Rf*: *Ruminococcus flavefaciens*, OAA: OxAloAcetate, PEP: phosphoenolpyruvate, Mal: malic acid, α-KG: α-ketoglutarate, and PDH: Pyruvate Dehydrogenase Complex.

Mitochondria, known as the “power station” of the cell, serve as the primary energy source for hepatocytes. Sufficient mitochondrial function in hepatocytes can be maintained through mitochondrial proliferation, the increased activity of key enzymes, and the elevated expression levels of key genes [47]. Citric acid, an important product of the tricarboxylic acid cycle, is formed by the condensation of CoA and oxaloacetate in the mitochondria. The tricarboxylic acid cycle provides pyruvic acid for mitochondrial metabolism. *P*yruvic acid, as one of its intermediate products, can enter the mitochondria during cellular respiration to participate in the tricarboxylic acid cycle [48]. In this study, no significant differences were observed in pyruvate and citric acid levels between Tibetan sheep and Hu sheep. As key intermediates involved in energy metabolism and nutrient cycling, the levels of citric acid and pyruvate can reflect the metabolic activity and energy supply within the cell, suggesting that the metabolic state of both is relatively stable, and further suggesting that the results of the present experiment are basically unaffected by rearing conditions and dietary status. Under aerobic conditions, mitochondrial oxidative phosphorylation serves as the primary source of ATP production in hepatocytes [49]. In this study, the ATP content in Hu sheep was significantly higher than that in Tibetan sheep. ATP was significantly and positively correlated with *Succiniclasticum* etc. Energy metabolism in ruminants is unlike that of monogastric animals, whose energy is mainly provided by the fermentation of microbiota in the rumen [36]. VFAs produced by rumen fermentation are absorbed by colonocytes and then enter the tricarboxylic acid cycle of mitochondria to provide energy for cellular ATP production. FHTD represents the final and rate-limiting step in the respiratory chain and serves as the regulatory hub of OXPHOS [50]. In this study, the activity of FHTD in Tibetan sheep was significantly higher than that in Hu sheep. This indicates that when adapting to the plateau environment, Tibetan sheep mainly rely on enzyme-catalyzed mitochondrial OXPHOS to supply energy. Additionally, mitochondrial organelles exhibit high plasticity and are involved in dynamic processes including mitochondrial fusion and fission, mitochondrial autophagy, and mitochondrial biogenesis [51]. These active processes occur continuously and simultaneously, primarily mediated by nuclear genes such as *Mfn1*, *Mfn2*, *OPA1*, *Fis1*, and *MFF*, which act on the mitochondrial membrane. In this study, the expression levels of fusion genes *Mfn1* and *Mfn2* and fission genes *Fis1* and *MFF* in Tibetan sheep were significantly higher than those in Hu sheep, and these genes showed a positive correlation with rumen microbiota such as *Ruminococcus albus*, *Selenomonas ruminantium*, and the *Rikenellaceae-RC9-gut-group*. This study found that rumen bacterial metabolites such as VFAs can serve as energy sources for mitochondria and influence their fusion and fission processes. The balance between mitochondrial fusion and fission determines mitochondrial morphology and enables their adaptation to cellular metabolic demands [52]. This indicates that the rumen microorganisms and the genes related to mitochondrial dynamics in Tibetan sheep work together to regulate the normal morphology of mitochondria so as to ensure the healthy growth of the organism. In addition, *PGC*-*1α*, as a major regulator of mitochondrial biogenesis, is important for the regulation of antioxidant defenses as well as mitochondrial network dynamics and autophagy removal from damaged mitochondria [53], while proteins encoded by the mitochondrial *ATP6* and *Cytb* genes play a role in cellular energy metabolism by participating in the process of energy conversion in the oxidative phosphorylation machinery [54,55]. We found that the expression levels of *ATP6*, *Cytb*, and *PGC*-*1α* in Tibetan sheep were all significantly higher than those in Hu sheep, and *PGC-1α* and *Cytb* were significantly and positively correlated with *Ruminococcus albus*, *Selenomonas ruminantium*, *Rikenellaceae-RC9-gut-group*, etc. The production of VFAs by these rumen flora, such as acetate and butyrate salts, can affect immune homeostasis and mitochondrial physiology through a variety of pathways [56], suggesting that the abundance of rumen flora directly affects mitochondrial functions. The functional state of the mitochondria also affects the composition and activity of the rumen microbiota. Mitochondrial dysfunction can lead to disturbances in the rumen metabolic environment, thereby affecting the growth and reproduction of rumen microbes. It is hypothesized that Tibetan sheep adapt to the hypoxic plateau environment by enhancing mitochondrial oxidative phosphorylation activity, the expression of mitochondrial dynamics-related genes, and mitochondrial biogenesis regulatory factors, while Hu sheep rely on the metabolic advantages of VFAs mediated by rumen microbiota to achieve efficient ATP production and maintain health (Figure 7).

## 5. Conclusions

Under the same feeding and management conditions, Tibetan sheep and Hu sheep exhibit distinct adaptation mechanisms to the Qinghai–Tibet Plateau environment. Tibetan sheep have a higher abundance of crude fiber-degrading bacteria such as *Ruminococcus albus*, *Ruminococcus flavefaciens*, *Selenomonas ruminantium*, *Clostridium butyricum*, and *Butyrivibrio fibrisolvens*, enabling them to strengthen crude fiber digestion and efficiently obtain energy from low-quality forage. In contrast, Hu sheep have a higher density of *Ruminobacteramylophilus*, *Treponema bryantii*, and *Fibrobacter succinogenes*, which more effectively digest proteins, starches, etc. They exhibit superior propionate production capacity and hepatic PC activity, thereby enhancing gluconeogenic precursor supply to maintain energy homeostasis. Tibetan sheep compensate for an insufficient propionate supply by upregulating PEPCK and FBPase activities, accelerating gluconeogenic intermediate metabolism to meet energy demands under hypoxic conditions. Additionally, Tibetan sheep show upregulated expressions of *PCK1*, *PCK2*, and mitochondrial dynamics-related genes *Mfn1*, *Mfn2*, *Fis1*, and *MFF*, along with increased FHTD activity and expressions of *ATP6*, *Cytb*, and *PGC-1α*, reflecting their maintenance of energy homeostasis under hypoxia through enhanced oxidative phosphorylation and mitochondrial biogenesis. In contrast, Hu sheep focus on the *FOXO1*-mediated regulation of gluconeogenic genes and ATP production via rumen microbiota-driven VFA metabolism. Microorganisms such as *Ruminococcus albus* and the *Rikenellaceae*-*RC9*-*gut*-*group* are positively correlated with gluconeogenic genes *G6PC1*, *PCK1*, and mitochondrial regulatory factors, highlighting the interactive role of microorganism–host in metabolic adaptation. These findings indicate that Tibetan sheep have formed strategies to adapt to high-altitude environments through rumen microbiota-mediated fiber degradation, the efficient utilization of gluconeogenic intermediates, and mitochondrial OXPHOS capacity. For the introduction of Hu sheep to high-altitude areas, dietary intervention may be required to regulate rumen microorganisms, such as enriching cellulose-decomposing bacteria or enhancing mitochondrial oxidative capacity, to counteract hypoxic-induced metabolic limitations.

## Figures and Tables

**Table 1:** Liver gluconeogenesis-related gene primers’ information.

Gene	Primers (5′–3′)	Length	Annealing Temperature	ID
*FBP1*	F: CCAGCTGCTCAACTCGCTTT R: CCAGCTATTCCATAGAGGTGCG	90 bp	60 °C	XM_004004092.5
*G6PC1*	F: GCGGCTGAACACAAAGGGAA R: AAGGTAGCGCCCAAAGTTGT	135 bp	60 °C	XM_012186137.4
*PCK2*	F: GCGGCTGAACACAAAGGGAA R: AAGGTAGCGCCCAAAGTTGT	84 bp	60 °C	XM_015096868.3
*FOXO1*	F: TCAGTCAACATCCGCAGTCA R: CAGGCGGTTCATACCCGAG	82 bp	60 °C	XM_027973596.2
*PCK1*	F: GGGAGTTCGTGGAGAGTAGC R: GCCTCTTGATCACACCCTCC	123 bp	60 °C	XM_004014441.5
*PGC-1α*	F: AGCTCCACGACTCCAGACA R: GTCGGAATCTGTGGAAGAGTGT	86 bp	60 °C	JF449960.1
*β-acting*	F: AGCCTTCCTTCCTGGGCATGGA R: GGACAGCACCGTGTTGGCGTAGA	113 bp	60 °C	NM_001009784

Note: *FBP1: fructose-bisphosphatase 1, G6PC1: Glucose-6-phosphatase catalytic subunit 1, PCK2: phosphoenolpyruvate carboxykinase2, FOXO1: Forkhead box O1, PCK1: phosphoenolpyruvate carboxykinase1, PGC-1α: Peroxisome proliferator-activated receptor-gamma coactivator-1-alpha*.

**Table 2:** Mitochondrial-related gene primers’ information.

Gene	Primers (5′–3′)	Length	Annealing Temperature	ID
*Mfn1*	F: TGGGCATCATCGTTGTTGGA R: AAAGGCTCTCTCCTTGGCAC	137 bp	60 °C	XM_004003134.5
*Mfn2*	F: ATGAACTGCACCGCCACATA R: TTGAGGTCGTAGCTGAGGGA	196 bp	60 °C	XM_004013714.5
*Fis1*	F: TGAAGTATGTGCGAGGGCTG R: CCATGCCCACTAGTCCATCTTT	108 bp	60 °C	XM_027961118.1
*MFF*	F: TCCAGCACGTGCATACTGAG R: CCGCCCCACTCACTAAATGT	107 bp	60 °C	XM_027965256.1
*ATP6*	F: GCCTCCTACCCCACTCATTTAC R: GGTGTCCCTTGTGGTAGGAAA	145 bp	60 °C	KT750051.1
*Cytb*	F: ACCCACTTAACACTCCCCCT R: GAGAGGATTAGGGCGAGGAC	112 bp	60 °C	FR873152.1
*OPA1*	F: CAGTTAAGGACGTCATTGCAGC R: CTGGCCAAAAATTCCTGTGGG	112 bp	60 °C	XM_060404590.1
*TFAM*	F: ATGGAAGTTGGACGAGAAGACC R: AAAGCTGCTCAGGCTCACTT	73 bp	60 °C	XM_027962472.2
*β-actin*	F: AGCCTTCCTTCCTGGGCATGGA R: GGACAGCACCGTGTTGGCGTAGA	113 bp	60 °C	NM_001009784

Note: *Mfn1: Mitofusin-1, Mfn2: Mitofusin-2, OPA1: Optic atrophy, Fis1: Mitochondrial fission 1, Mff: Mitochondrial fission factor, ATP: Subunit 6 of ATP synthase, Cytb: Cytochrome b, and TFAM: Mitochondrial transcription factor A*.

**Table 3:** Rumen microbiota primers’ information.

Gene	Primers (5′–3′)	Length	Annealing Temperature	ID
*Bf*	F: CCTGACTAAGAAGCACCGGC R: GTAAAACCGCCTACGCTCCC	107 bp	60 °C	U41167.1
*Ra*	F: GGGCTTAACCCCTGAACTGC R: TCGCCACTGATGTTCCTCCT	114 bp	60 °C	X85098.1
*Ram*	F: GGGGACAACACCTGGAAACG R: CTTGGTAGGCCGTTACCCCA	124 bp	60 °C	Y15992.1
*Sr*	F: AGAAAGCCACGGCTAACTAC R: TCTCCTGCACTCAAGAAGAC	169 bp	60 °C	AB198442.1
*Tb*	F: ATGGCAGGTACAGAGTGAAG R: TTCAAGGAGTCGGGTTTCAG	95 bp	60 °C	NR-118718.2
*Rf*	F: CTAATCAGACGCGAGCCCAT R: ACATGCAAGTCGAACGGAGT	196 bp	60 °C	LT976286.1
*Fs*	F: GATGAGCTTGCGTCCGATT R: ATTCCCTACTGCTGCCTCC	110 bp	60 °C	EU606019.1
*Cb*	F: CATTGGGACTGAGACACGGC R: AAGACCGTCATCACTCACGC	108 bp	60 °C	NR_042144.1
*Bacterium*	F: CCTACGGGAGGCAGCAG R: TTACCGCGGCTGCTGG	181 bp	60 °C	*

* indicates that the bacterium was the steward gene sequence (the 16S rRNA sequence). Note: *Ra: Ruminococcus albus, Ram: Ruminobacteramylophilus, Sr: Selenomonas ruminantium, Tb: Treponema bryantii, Cb: Clostridium butyricum, Fs: Fibrobacter succinogenes, Bf: Butyrivibrio fibrisolvens, and Rf: Ruminococcus flavefaciens*.

## Data Availability

The data sets presented in this study can be found in the NCBI Sequence Read Archive (SRA) under accession numbers PRJNA1135557.

## Supplementary Materials

The following supporting information can be downloaded at: https://www.mdpi.com/article/10.3390/ani15111603/s1, Table S1: Nutrient composition of forage in Gannan; Table S2: Concentrations of VFAs in the rumen of Tibetan sheep and Hu sheep.
