# Supplementary material for: A Study on the Differences in Rumen Microbiota–Liver Gluconeogenesis–Mitochondrial Interaction Between Tibetan Sheep and Hu Sheep in the Qinghai–Tibet Plateau
Source: Animals (Basel). 2025 May 30;15(11):1603. doi: 10.3390/ani15111603 (PMC12153551; doi:10.3390/ani15111603)
Supplement: Supplementary file 1 [file animals-15-01603-s001.zip › animals-3623892-supplementary.pdf]

Table S1. Nutrient composition of forage in Gannan

Indicators/Periods	*Poa poophagorum Bor*	*Poaceae*	*Carex coninux*	*Argentina anserina*	*Geranium platyanthum Duthie*
Aboveground biomass (g/m^2)	455.30	565.00	463.20	454.30	484.50
Height (cm)	18.80	21.00	21.50	19.30	18.50
Dry matter (%)	94.53	94.70	94.48	94.64	94.69
Crude protein (%)	11.33	11.28	10.84	11.54	11.27
Ether extract (%)	4.57	4.16	4.05	4.24	4.47
Crude Ash (%)	7.37	7.29	7.42	7.32	7.36
Neutral detergent fiber (%)	58.37	59.03	58.45	57.43	57.13
Acid detergent fiber (%)	34.23	34.57	33.81	33.69	33.58
Ca (%)	0.88	0.85	0.95	0.82	0.95
P (%)	1.17	1.69	1.30	0.96	0.91

Table S2. Concentrations of VFAs in the rumen of Tibetan sheep and Hu sheep

position	Item	Hu sheep	Tibetan sheep	*P* value
Rumen	Acetate/（mmol/L）	30.191±1.682	21.939±0.898	0.002
	Propionate/（mmol/L）	6.118±0.233	3.392±0.214	0.000
	Isobutyrate/（mmol/L）	0.198±0.027	0.436±0.049	0.002
	Butyrate/（mmol/L）	2.412±0.332	1.903±0.025	0.057
	Isovalerate/（mmol/L）	0.239±0.061	0.612±0.035	0.001
	Valerate/（mmol/L）	1.162±0.027	0.643±0.015	0.000
	Total VFAs/（mmol/L）	40.321±1.319	28.926±0.869	0.000
	Acetate/Propionate（A/P）/（mmol/L）	4.9436±0.415	6.4924±0.628	0.024
